# Fission-fusion dynamics over large distances in raven non-breeders

**DOI:** 10.1038/s41598-017-00404-4

**Published:** 2017-03-23

**Authors:** Matthias-Claudio Loretto, Richard Schuster, Christian Itty, Pascal Marchand, Fulvio Genero, Thomas Bugnyar

**Affiliations:** 1 Department of Cognitive Biology, University of Vienna, Althanstrasse 14, A-1090 Vienna, Austria; 2 Konrad Lorenz Forschungsstelle, Core Facility for Behaviour and Cognition, University of Vienna, Fischerau 11, A-4645 Grünau im Almtal, Austria; 3 Department of Forest and Conservation Sciences, University of British Columbia, 3041 – 2424 Main Mall, Vancouver, B.C. V6T 1Z4 Canada; 4 Department of Biology, 1125 Colonel By Drive, Carleton University, Ottawa, K1S 5B6 Canada; 5 Office National de la Chasse et de Faune sauvage, Délégation Régionale Occitanie, Actisud Bâtiment 12, 18 rue Jean Perrin, F-31100 Toulouse, France; 6 Riserva naturale regionale del Lago di Cornino, I-33030 Forgaria nel Friuli, Udine, Italy

## Abstract

The influence of fission-fusion dynamics, i.e., temporal variation in group size and composition, on social complexity has been studied in large-brained mammals that rely on social bonds. Little is known about birds, even though some species like ravens have recently received attention for their socio-cognitive skills and use of social bonds. While raven breeders defend territories year-round, non-breeders roam through large areas and form groups at food sources or night roosts. We here examined the fission-fusion patterns of non-breeding ravens over years, investigating whether birds meet repeatedly either at the same or at different locations. We combined four large datasets: presence-absence observations from two study sites (Austria, Italy) and GPS-tracking of ravens across two study areas (Austria, France). As expected, we found a highly dynamic system in which individuals with long phases of temporary settlement had a high probability of meeting others. Although GPS-tagged ravens spread out over thousands of square kilometres, we found repeated associations between almost half of the possible combinations at different locations. Such a system makes repeated interactions between individuals at different sites possible and likely. High fission-fusion dynamics may thus not hinder but shape the social complexity of ravens and, possibly, other long-term bonded birds.

## Introduction

Life in structured social groups poses challenges^1, 2^, that affect individuals’ fitness^3, 4^ and may drive brain evolution^5, 6^. In support of this hypothesis, pair-bonded species across several taxonomic groups of mammals and birds have larger brains than their relatives with different breeding systems, indicating a key role of relationship quality between particular individuals^7, 8^. In primates, the forebrain size correlates positively with group size rather than with pair bonding^9^, which is in line with the finding that primates can deal with different types of valuable relationships simultaneously^10^. Another factor contributing to social complexity may be seen in a species’ fission-fusion dynamics, i.e., the extent of changes in group cohesion and individual membership over time^11^. It has been proposed that high degrees of fission-fusion dynamics require a high flexibility in dealing with social relationships and may go together with improved memory, inference of e.g., ranks and inhibition skills^11^. Mammalian species with large brains compared to their body size (e.g., apes, dolphins, elephants) tend to combine these complexity factors: they live in fairly large groups with differentiated social relationships and relatively high degrees of fission-fusion dynamics^11–14^ but also in bat colonies long-term social relationships have been found^15^. Although large-brained birds do form differentiated social relations like partnerships, their group sizes tend to depend on several external and internal factors and flexibly change with the availability of environmental resources, breeding status and season^16^. However, this flexibility in forming groups does not mean that flocks are simply anonymous crowds that aggregate at resources. In fact, flocks may consist of different social layers, with familiar and/or related individuals forming sub-groups (e.g., greylag geese, *Anser anser* ^17^) or other temporarily stable units, such as groups with different foraging modes in ravens^18^. Here we investigate the question whether bird flocks can be characterized by fission-fusion dynamics that are comparable with those of some mammals, i.e., given individuals repeatedly meet at different sites.

Common ravens (*Corvus corax*) attract attention for their advanced socio-cognitive skills^19^. Notably, their life as non-breeders appears to be socially challenging: they often form groups during foraging^20, 21^ and actively recruit others in order to overcome the food defence of territorial breeding pairs^22^. Even though non-breeding ravens compete heavily with each other for access to food and for keeping their food caches^23, 24^, outside of feeding events they also play, socialize and roost together^25^. Individuals thereby engage in various affiliative interactions, which may result in the formation of social bonds, which is advantageous in conflicts^26–28^: bonded birds tend to support each other in fights^29, 30^, they win more conflicts than non-bonded birds and obtain high dominance status^26, 27^; bonding partners also engage in forms of post-conflict affiliation^31, 32^, indicating sophisticated relationship repair and support mechanisms. Captive ravens remember their relationship valence to former group members up to three years after being separated from them^33^ and they notice dominance reversals among conspecifics within and outside of their social group^34^.

To sum up, ravens frequently form groups of different size and composition. They rely on social bonds and show socio-cognitive skills that are in many aspects comparable to those of primates. These findings suggest that despite the fluid character of raven groups, individuals repeatedly meet and interact with each other. In a study by Heinrich *et al*.^35^ ten ravens were trapped next to a temporary food source and radio-tagged, but the results did not support the idea of birds meeting each other at different locations. Furthermore, the degree of kinship in raven non-breeder groups was found to be very low^36^. However, juvenile ravens tend to stay close to their siblings during dispersal^37^ and in a previous study we found that non-breeding ravens of several age classes may develop local preferences for using anthropogenic food sources^27^. There were high levels of vagrancy by other individuals at the study site and recent results show that these vagrant ravens use a variety of anthropogenic food sources^38^. Exploiting anthropogenic food sources has a long history in ravens and overall corvid and human remains are commingled in settlements up to 10,500 years old^39^. Without human influence, ravens also seem to have some kind of predictable food sources, e.g., when associating with wolf packs to scrounge food from their kills^40–42^.

Todays’ environment in Central Europe offers an ideal opportunity to study grouping patterns and fission-fusion dynamics of ravens, as individuals can be easily localized at anthropogenic foraging sites and food abundance seems not to be a limiting factor^38^. We tested the hypothesis that despite the fluid character of raven non-breeder groups, individuals come in repeated contact either by regularly using a single resource over a given time period (fission and fusion at a stable food source or night roost) and/or by meeting from time to time at different sites (fusion at site A, fission, fusion at site B). We investigated the individuals’ tendency to use temporarily stable food sources over longer time periods and quantified how this increased contact between particular birds. Furthermore, we investigated whether factors like age class, sex or breeding status affect this tendency. Since all these factors are linked with dominance status^26^, we expected them to influence how consistently individuals use a certain food source and hence repeatedly meet other ravens.

We analysed four large datasets: presence-absence observations from two study sites (Austria, Italy) to calculate the percentage of co-occurrence of each individual combination at both sites and GPS-tracking of ravens across two study areas (Austria, France).

## Results

### Local patterns: use of a single food source and chances of repeatedly meeting others

At the Austrian study site (AUT), 185 out of 256 marked ravens were observed at least once co-feeding with the zoo animals during the study period of 4.5 years (1091 observation days). From these 185 birds, 126 (68%) reached our threshold of being seen at least ten times; most individuals were ﻿visiting the site regularly (mean number of observations per bird = 207.8; Table 1). At the Italian study site (ITA), where carcasses are continuously provided for vultures 73 out of 76 marked ravens have been seen at least once during 4.25 years (662 observation days). 52 (71%) of these were present at least on ten days (mean number of observations = 87.5; Table 1). Typically, periods of presence (AUT: mean = 3.3 days, max = 85 days; ITA: mean = 1.7 days, max = 19 days) alternated with periods of absence (AUT: mean = 22.5 days, max = 503 days; ITA: mean = 31.8 days, max = 528 days; Table 1). Overall, individuals spent significantly longer time periods absent than present (Wilcoxon signed-rank test, AUT: *N* = 185, *V* = 2813.5, *P* < 0.001, ITA: *N* = 73, *V* = 29, *P* < 0.001). As expected we observed large variation between individuals and sites in how often birds were present at the food sources (Supplementary Fig. S1a,b): in Austria 21% of the birds could be seen on more than 2/3 of the observation days, meeting our definition as “locals”, 35% of the birds were categorized as “frequent visitors” (present between 1/3 and 2/3 of the observation days) and 44% as “rare visitors” (present on less than 1/3 of the observation days). At the Italian study site only one individual could be classified as local, 17% of the birds as frequent visitors and 81% as rare visitors. Note that the food source in Italy is available all day and year-round, while the food in Austria is present during a short time period in the morning only.

**Table 1:** To illustrate the individual variation we calculated for the Austrian and Italian study site mean, minimum and maximum values over all individuals for: the number of consecutive days being present or absent, the total number of days present and the percentage of days present (data refer to the time between marking and their last observation).

	# of consecutive days present	# of consecutive days absent	# of days present^1^	% of all possible days present^1^
Austria	3.3 (1–85)	22.5 (1–503)	207.8 (11–973)	40.7% (0.03–92.9%)
Italy	1.7 (1–19)	31.8 (1–528)	87.5 (10–284)	22.6% (0.02–69.2%)

^1^Only individuals with at least 10 observations were included; N = 126 for AUT and N = 52 for ITA.

**Figure 1:**
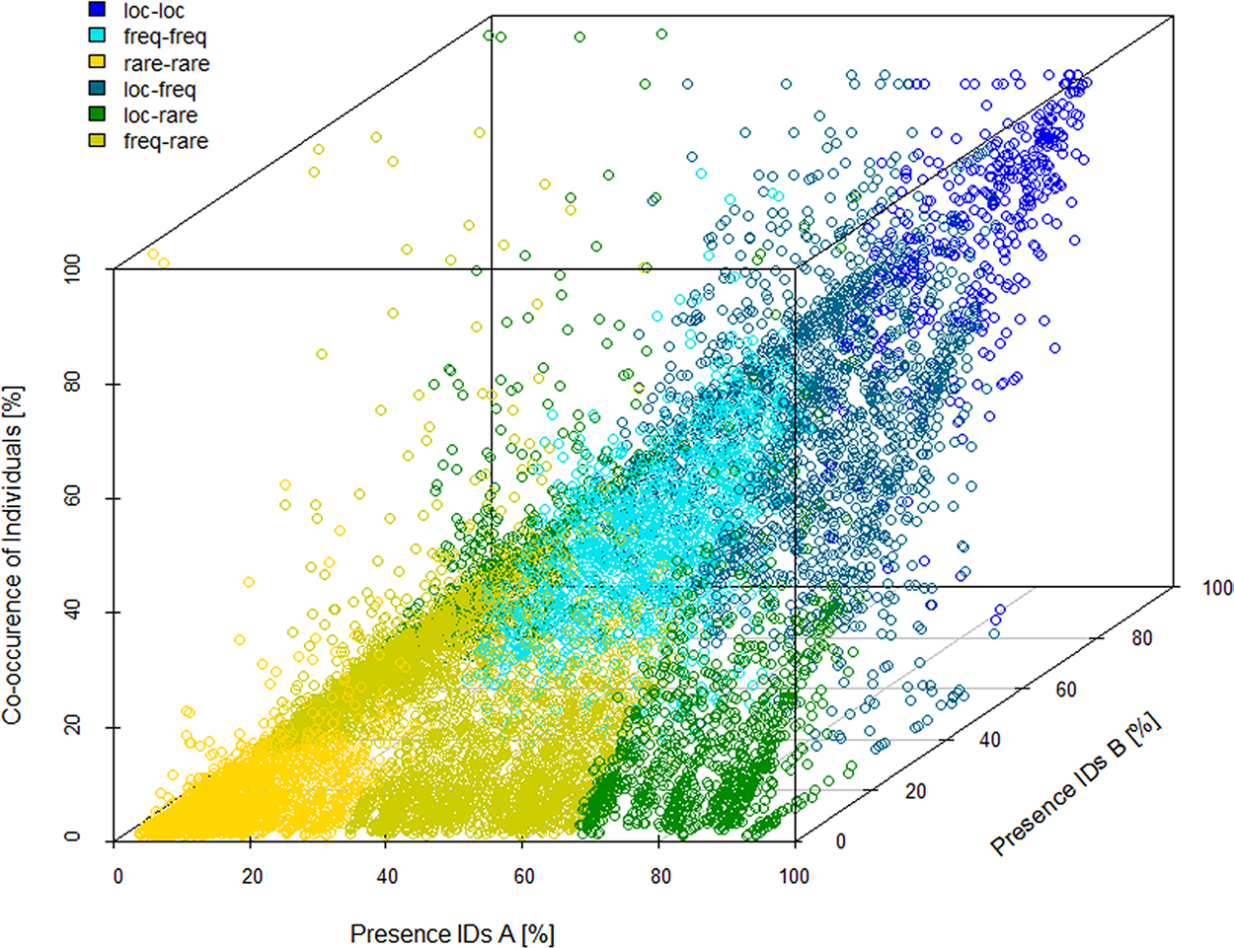
Co-occurrence of every combination of two individuals (A + B) at a single food source (Austrian study site). The percentage of days each raven was present is always shown for one individual of each combination on the x-axis (IDs A) and for the other one on the y-axis (IDs B). The z-axis represents the percentage of co-occurrence of these individuals. The different colors show the combinations of classified presence patterns: locals (loc), frequent visitors (freq) and rare visitors (rare). Only ravens that were present at least 10 times are included (N = 126).

Out of 12,206 dyadic combinations (i.e., combinations of two individuals) in Austria local ravens encountered other locals on average on 69.6% of the days, while frequent visitors co-occurred with each other in 30.1% and rare visitors with other rare visitors only in 2.1% (Table 2 and Fig. 1). For the Italian site (2,414 dyadic combinations) the comparison between locals could not be made, since only one individual was classified as local. However, all other values reflect the same pattern as in the Austrian dataset (Table 2).

**Table 2:** For both study sites (Austria, Italy) the mean percentage of co-occurrence between individuals of different classes is shown: locals (loc), frequent visitors (freq), rare visitors (rare).

	loc-loc	loc-freq	freq-freq	loc-rare	freq-rare	rare-rare
Austria	69.6	43.8	30.1	11.5	7.7	2.1
Italy	NA	46.1	25.7	22.5	10.9	5.0

Data refer to the time between marking and their last observation.

None of the models with possible combinations of the factors sex, breeding status (breeders versus non-breeders) and estimated age at the first observation improved penalized model fit over the null model, as assessed by AIC, indicating that variation in the data cannot be explained by any of these factors^43^. Contrary to our expectation, indicators of dominance do not predict how consistently ravens use a site and meet other individuals.

### Large scale patterns: Fission and fusion at different sites

In the time period of up to 45 months in the Austrian Alps nine individuals were tracked with GPS-loggers that measured their position with a minimum interval of one hour. Thus, we could calculate the distance between all 36 dyadic combinations of individuals, where the positions of both individuals were measured (59,082 calculated distance values); in the French population during a time period of up to 20 months 18 individuals were tracked with the GPS-loggers and led to n = 126 dyadic combinations and 206,355 distance calculations (27 of the possible dyadic combinations had to be excluded since individuals were not GPS-tagged at the same time). We then filtered only those occurrences when individuals stayed within 100 m (AUT: 1,668; FRA: 5,004), which were previously defined as associations. In the Austrian Alps 15 dyadic combinations were found at least once within 100 m, ranging from 0.1 to 19.1% (mean 6.5%) of all distance calculations and every individual was included at least once (Supplementary Table S3). Despite the translocation of 8 individuals over distances up to 240 km in the French study area, 49 combinations were found at least once within 100 m, ranging from 0.3 to 30.6% (mean 10.3%) of all distance calculations. If we focus only on the non-translocated individuals 32 out of 39 combinations could be found within this short distance (Supplementary Table S4). These associations could often be detected over consecutive time steps (AUT: up to 7 hours, FRA: up to 5 hours) during the day, indicating that individuals may stay or move together for longer time periods.

**Figure 2:**
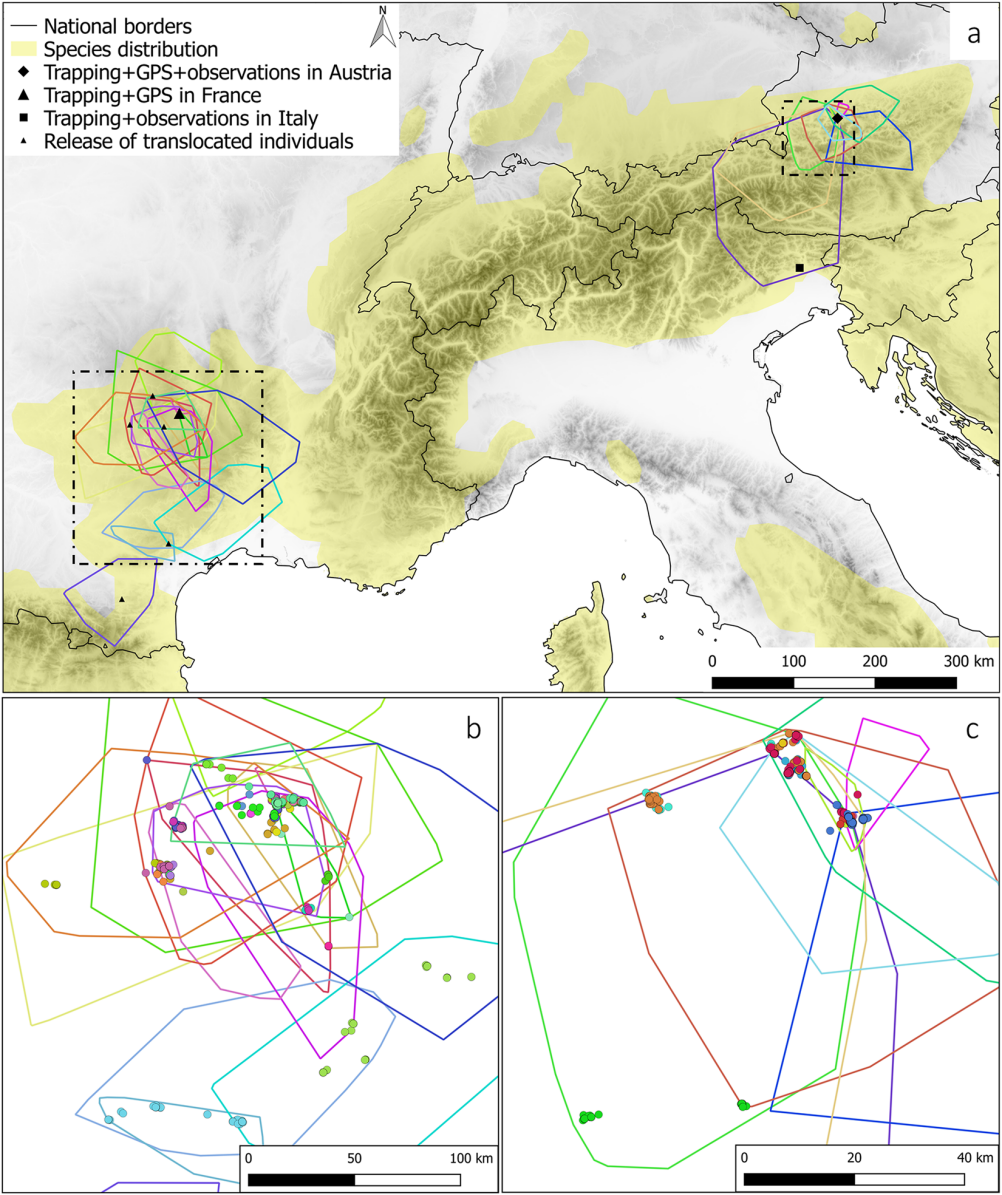
Fission-fusion dynamics of GPS-tagged ravens. (**a**) The three study areas are marked with a diamond for AUT, a square for ITA and a triangle for FRA. All individuals with GPS-tags have been captured and released in AUT or in FRA (small triangles indicate release points for translocated individuals). The minimum convex polygon (100%) of each individual is represented in different colors to roughly cover their space use. The dashed squares indicate the areas of (**b,c**), both of which show the GPS-locations as points (5,004 for FRA and 1,668 for AUT) of fusion events at different sites. The colors of the points indicate different combinations of ravens. The maps were created using QGIS 2.18.1, an Open Source Geospatial Foundation Project: http://qgis.osgeo.org, QGIS Development Team, 2016.

Note that GPS-tagged ravens spread out over thousands of square kilometres from the release sites (cf. ref. 38) and the locations of all dyadic associations were often more than 100 km away from each other (Fig. 2). In the Austrian study area we visited all these locations and we could always identify rich food sources (2 game parks, 2 compost stations, 2 skiing areas and 1 garbage dump) plus adjacent common night roosts within one hundred metres to several kilometres distance. At each food source, we observed a minimum of 30 to 50 ravens. In the French study area, we also visited some of these sites, which always showed the same pattern: many ravens used a rich and permanent food source and adjacent night roosts.

## Discussion

We described fission-fusion patterns of wild non-breeding ravens at different spatio-temporal scales and found that i) many birds came in repeated contact with each other on a daily basis at temporally stable food sources, and ii) they repeatedly met at several of these foraging sites which can be located >100 km away from each other.

Focusing on a given foraging site revealed a large variation in how often individuals are present and how long they stay, despite the high temporal consistency of the food sources (daily feedings of zoo animals in AUT and carcasses for vultures in ITA). A subsample of ravens, both breeders and non-breeders, seemed to specialize on these areas and could be observed almost daily over weeks, months or even years, whereas the majority of ravens visited these areas only occasionally. This is particularly true for the Austrian site, for which our dataset corroborates earlier results that were based on a much smaller sample over a shorter time period^27^. At the study site in Italy, presence-absence patterns were similar to those in Austria except for the absence of “local” individuals. This difference may be caused by the feeding regimes at the two sites, with zoo animals being fed during a relatively short time in the morning in Austria, whereas vultures were provided with carcasses throughout the day in Italy. In the latter case, ravens can access the food at any time, resulting in a rather low spatio-temporal coordination among individuals. In such a situation, it is hardly possible for human observers to monitor all birds throughout the day. When ravens can exploit a regular food supply only at certain times of the day as at the Austrian study site, the likelihood of fusion events is enhanced and individual birds are easier to spot.

As expected, ravens spending much time at one site have an increased chance of repeated encounters with the same conspecifics during foraging. This finding is apparently intuitive, but it is the first study that really quantifies the chances of repeated encounters of non-breeding ravens, and possibly non-breeding birds in general. From previous studies at the Austrian study site we know that ravens using the zoo feedings in the morning also tend to stay in that area during the rest of the day and gather at the adjacent night roost^44^. We suggest that these regular fusion events around a food source and its surrounding area enable the emergence of a local social structure with a linear dominance hierarchy and temporary stable affiliation patterns^27^. Note, that the majority of ravens repeatedly visited our study site for a few days but spent longer time periods away. They either wandered around, moving from one anthropogenic food source to the next, or they were locals at other foraging sites^38^. Such frequent or rare visitors at a foraging site showed a reduced probability to repeatedly meet other frequent or rare visitors, but still had a relatively high probability to repeatedly encounter the local birds. Thus, frequent visitors may be familiar at least with the social structure of locals in an area. Accordingly, we speculate that locals and frequent visitors base their interactions on detailed knowledge about the others’ relationships^34, 45^, whereas interactions between frequent and/or rare visitors may follow simpler rules like ‘avoid older birds’ or ‘always help the aggressor’.

In addition to the association patterns emerging around a single foraging site, we found that non-breeding ravens may also meet repeatedly over years at locations with similar characteristics even far more than 100 km apart from each other. To our knowledge, this is the first evidence of such kind of dynamics for non-migratory birds in the non-breeding phase (cf. ref. 16). Notably, it suggests that the social network of non-breeding ravens includes individuals on a small and large spatio-temporal scale.

The data from GPS-tagged individuals of two populations (AUT, FRA) support the presence-absence data at two study sites (AUT, ITA): ravens stayed in the vicinity of a rich and predictable food source for different time periods, during which they had a certain likelihood to repeatedly encounter other individuals using the same food source. The finding, that combinations of two GPS-tagged birds were often within 100 m at consecutive measured time steps over several hours, suggests, ravens met not only during foraging events and makes the occurrence of social interactions other than those associated with food competition likely. Indirect support for this interpretation comes not only from several observational studies (e.g., refs 21, 27, 46 and 47) and from our visits to locations, where GPS-tagged birds associated: we always found large raven groups (sometimes including our GPS-tagged individuals) during and outside of feeding events physically and acoustically interacting with each other. What we do not know so far is whether ravens which repeatedly met at different locations also interacted with each other more often and/or in different ways than they do with birds they met only at one place. Future studies may focus on the possibility of vagrant ravens forming social relationships among each other (cf. ref. 18).

On the basis of the current data (presence-absence data, GPS-tracking, observations at important food sources), we may roughly estimate the population size of non-breeding ravens in the Eastern Alps, i.e., how many conspecifics the GPS tagged ravens could theoretically meet. Focusing on the area represented in Fig. 2c, it includes a total of 7 food sources plus adjacent night roosts, where associations of GPS-tagged non-breeders have been found. Since we observed 30 to 50 ravens at these sites at each of our visits, we can expect several hundred individuals in this area. When taking into account the entire area used by our 9 GPS tagged birds (Fig. 2a), which includes many more food sources (cf. ref. 38), and the fact that 250 individuals were marked at only one of these sites, we can assume that many hundreds if not more than a thousand non-breeding ravens live in this part of the Alps (Fig. 2a). Even though captive ravens show remarkable abilities in remembering individuals and former relationships^33^, it is probably unrealistic that every raven in this population would (repeatedly) come in contact with and know all others. Hence, both patterns revealed in this study, i.e. forming local subgroups and repeatedly meeting at different locations, indicate ‘anchor structures’ within a highly fluid system that allows social relationships to develop.

Most ravens showed strong fission-fusion dynamics despite their opportunity to exploit food sources that are rich and stable over time (cf. ref. 38). This stands in contrast to many other social systems where changing availability of food or other resources causes these dynamics^48, 49^. Possibly, the ravens’ behaviour is caused by their scavenging life style with a predisposition to wander around and a high individual flexibility in developing site preferences. Despite our substantial data set from the Austrian study site, our expectation that factors indicative for social dominance (i.e., age class, sex or breeding status) would influence ravens’ resource use and thus their group dynamics is not supported. These results are in line with other studies, showing no difference between males, females and different age classes concerning the space use of non-breeding ravens^44, 50, 51^. Finding no effect of breeding status, however, might be an artefact of our sampling method at one location only. Since breeders defend a large territory year round^51, 52^ only few of them live close enough to use the food source daily, while others might use it only from time to time. Future studies should address other factors that could determine group dynamics like social integration, presence of kin and differences in temperament and personality, i.e., the birds’ propensity to form routines or to show exploration/avoidance (cf. ref. 53 and references therein). Further, a comparative approach with other scavenging birds would be highly interesting. Many vulture species might show similar fission-fusion dynamics as they aggregate at food sources and communal roosts e.g., Griffon vultures (*Gyps fulvus*)^54^ or Egyptian vulture (*Neophron percnopterus*)^55^. In the American black vulture (*Coragyps atratus*) not only close contact between family members have been observed throughout the year, but also preferred associations between certain families^56^. However, for most of these species it has not been studied whether their associations lead to repeated interactions and little is known about the social relationships of non-breeders and their socio-cognitive skills.

This study reveals a complex pattern of fission-fusion dynamics in non-breeding ravens that work on different spatio-temporal scales. Staying close to a stable food source leads to a high probability of interacting with given individuals that regularly visit the same location; repeated encounters at different sites that are several or many kilometres apart offers another opportunity for non-breeders to get to know each other. Repeated encounters and interactions are the basis for the social relationships and the evolution of socio-cognitive skills. We can therefore confirm that taking into account fission-fusion dynamics for studying the evolution of social cognition is a promising route for research not only in mammals but also in birds.

## Methods

### Study areas and data collection

#### We combined four datasets collected in Central Europe:

Presence-absence observations of individually marked ravens from two study sites (Austria and Italy) and GPS-tracking of ravens trapped at the same site in Austria and another site in France.

#### Study area in Austria (AUT)

Depending on the season, 15 (summer) to 120 (winter) wild ravens can be observed every day in the Cumberland Wildpark, a zoo (47.80°N, 13.95°E) situated in a narrow valley at the northern edge of the Alps. Non-breeders as well as several breeding pairs (estimated number 7–12) use this park as a regular food source by scrounging at the feedings of captive animals such as brown bears, wolves and wild boars. Importantly, the zoo animals are fed every morning between 8 and 10 am, making the food available for ravens only for a short time period of the day. 256 wild ravens were caught in drop-in traps and individually marked with coloured rings and patagial wing tags in the years 2007 to 2013. We took blood samples for sexing and estimated their age based on their mouth and feather colouration^57, 58^. Breeders are defined as birds defending a territory for reproduction; throughout the year, they can be distinguished from non-breeders by their high rates of aggressive and self-aggrandizing behaviours^59^. During 2011 and 2015 the presence of marked individuals has been monitored at least every second day at the feedings in the zoo. Observations were made at the enclosures of wild boars, wolves and bears for about 20 minutes per site, resulting in around 60 minutes observation time per day and more than 1000 hours in total. Individual ravens were identified by their coloured rings and wing tags. In 2013 we outfitted 9 non-breeding ravens with solar powered GPS-loggers mounted as backpacks (model Duck 4 C, Ecotone Telemetry, Poland; www.ecotone.pl; for details see also ref. 38), never exceeding 3% of the bird’s body weight^60^. Since ravens are diurnal birds, we intended to get a GPS position every full hour starting before sunrise until after sunset to also include the position of the night roost. The GPS fixes varied between individuals and time of the year, since some loggers did not perform as well as others and bad light conditions, especially during short winter days, required larger sampling intervals of several hours; yet, we could use GPS data of 9 individuals from March 2013 to end of November 2016 (Supplementary Table S1).

#### Study area in France (FRA)

Up to approximately 200 ravens can be observed at a garbage dump in Saint Flour, a city in the Massif Central in the middle of Southern France (45.05°N, 3.10°E). With the same methods as described above we caught non-breeding ravens and outfitted 18 individuals with GPS-loggers, coloured rings and wing tags. While 10 individuals were released at the trapping site, 8 ravens were translocated over distances of up to 240 kilometres as part of management operations aiming to reduce raven abundance in the surroundings of the garbage dump. In the results section we explicitly indicate the translocated individuals; see also Supplementary Table S2. The data were collected from April 2015 until end of November 2016 with the same sampling interval as described above.

#### Study area in Italy (ITA)

Within the nature reserve “Lake of Cornino” a permanent feeding station for wild griffon vultures (*Gyps fulvus*) has been established, where beside vultures up to 100 ravens come to feed every day. From 2000 to 2003, we caught 76 ravens with drop-in traps and marked them individually with coloured rings. In combination with a monitoring for individually marked griffon vultures (*Gyps fulvus*) the presence of marked ravens was recorded between May 2001 and July 2005 but less frequently than in Austria. Note that in contrast to the Austrian study site, food is provided at the Italian study site throughout the day. This leads to a large and unpredictable variation in the number of ravens (and also vultures) present during each day. Instead of using predefined times to record the presence of marked individuals, we focused on periods of high activity for around 15–30 minutes on each observation day. For this dataset we had no information about sex, territoriality (breeder versus non-breeder) or minimum estimated age.

### Analysis of presence-absence data

We analysed presence-absence data of marked ravens collected on 1091 days between 1st January 2011 and 30th June 2015 at the Austrian study site and on 662 days between 1st May 2001 and 31st July 2005 at the Italian study site. For all individuals in each dataset we calculated mean, minimum and maximum values for: the number of consecutive days each marked raven was present or absent, the total number of days it was present and the percentage of days present. Note, that for every individual we only refer to the time between marking and their last observation, since afterwards we did not know whether the raven was dead or just left the area. Ravens that were present more than 2/3 of the days were classified as “locals”, those individuals present less than 1/3 of the timespan as “rare visitors” and all others as “frequent visitors”. For every dyadic combination we calculated the percentage of days both individuals were present out of all possible days when they could have been present together (i.e., the overlap of observation days, when both individuals were already marked and before they had been observed for the last time, % co-occurrence). Finally, for data from the Austrian study site we tested whether sex, territoriality (breeders versus non-breeders) or minimum estimated age at the first observation could explain the average number of consecutive days present or absent and the percentage of being present using generalized linear models (error distribution = beta, link = logit).

### Analysis of GPS data

For the ravens outfitted with GPS loggers (9 in Austria and 18 in France), we measured for each study area the distance between all individuals at every possible time step. One time step was defined as one hour, the shortest interval of GPS-fixes during daytime, usually measured at every full hour. If a time step of an individual from a combination was missing due to e.g., low battery level or deviated more than 10 minutes, this time step was not used in the subsequent analysis. Thus, we only used the positions of all dyadic combinations in each study area during the time when both individuals were outfitted with active GPS-loggers. We defined an association between two individuals to be, when their Euclidean distance at the same time was less than 100 m. The percentage of these occurrences out of all possible time steps was calculated as well as their maximum duration, i.e., number of consecutive time steps within a distance of 100 m.

To identify the areas where individuals met over time, we plotted their locations on a map using the software R^61^ and QGIS 2.18.1 (QGIS Development Team, 2016). Subsequently, in Austria we visited these sites to investigate whether ravens other than our GPS-tagged birds use these areas and to describe their environmental characteristics that might have led to raven aggregations, for example the presence of foraging sites like garbage dumps or night roosts (the latter could also be determined by GPS fixes between dusk and dawn).

#### Ethics

All procedures performed in this study involving animals were in accordance with the ethical standards of the Austrian, French and Italian government guidelines and the institutional guidelines of the University of Vienna. Specifically, the study was approved by the Internal Ethics Committee (Permit Number 2014–018) of the Faculty of Life Sciences, University of Vienna and the Austrian Ministry of Science, Research and Economy (Animal Experimentation Permit Number 66006/0019-WF/II/3b/2014, received by TB). Additional permissions for trapping and GPS-tagging in France: Centre de Recherches sur la Biologie des Populations d’Oiseaux, No. 010656101028; and for trapping and marking in Italy: ISPRA (Istituto Superiore per la Protezione e Ricerca Ambientale).

## Electronic supplementary material

Supplementary Information
