# Supplementary material for: Fission-fusion dynamics over large distances in raven non-breeders
Source: Sci Rep. 2017 Mar 23;7:380. doi: 10.1038/s41598-017-00404-4 (PMC5428508; doi:10.1038/s41598-017-00404-4)
Supplement: Supplementary file 1 — Supplementary Information [file 41598_2017_404_MOESM1_ESM.pdf]

Supplementary Information for

Fission-fusion dynamics over large distances in raven non-breeders

Matthias-Claudio Loretto[1,2*], Richard Schuster[3,4], Christian Itty[5], Pascal Marchand[5], Fulvio Genero[6], Thomas Bugnyar[1,2]

[1] Department of Cognitive Biology, University of Vienna, Althanstrasse 14, A-1090 Vienna, Austria

[2] Konrad Lorenz Forschungsstelle, Core Facility for Behaviour and Cognition, University of Vienna, Fischerau 11, A-4645 Grünau im Almtal, Austria

[3] Department of Forest and Conservation Sciences, University of British Columbia, 3041 – 2424 Main Mall, Vancouver, B.C. Canada V6T 1Z4

[4] Department of Biology, 1125 Colonel By Drive, Carleton University, Ottawa K1S 5B6, Canada

[5] Office National de la Chasse et de Faune sauvage, Délégation Régionale Occitanie, Actisud Bâtiment 12, 18 rue Jean Perrin, F-31100 Toulouse, France

[6] Riserva naturale regionale del Lago di Cornino, I-33030 Forgaria nel Friuli, Udine, Italy

*Corresponding author

E-mail: mail@m-loretto.com

This file includes: Table S1

Table S2

Table S3

Table S4

Figure S1 a, b

Table S1: General information about GPS-tagged non-breeding ravens (ID) trapped in Austria. All individuals were captured on different days. The data collection for most ravens already stopped before end of November 2016 due to (i) technical reasons (individuals were observed with the GPS logger but no data were transmitted), (ii) removal of the GPS-logger by the raven in one case and (iii) predation in another case.

ID	fledged in	sex	date of tagging	last GPS fix	# days with data	# GPS fixes	mean # of fixes per day
**C8**	2011+	f	20/04/2013	13/04/2016	600	3604	6.0
**C5**	2012	f	29/03/2013	07/10/2016	651	2784	4.3
**C6**	2012	f	14/04/2013	30/11/2016	1317	12753	9.7
**C10**	2013	f	01/09/2013	24/04/2014	233	1937	8.3
**C21**	2012+	m	16/11/2013	26/11/2016	1055	11483	10.9
**C11**	2012	m	03/09/2013	27/07/2016	932	7190	7.7
**C12**	2012	m	31/10/2013	20/05/2015	536	5106	9.5
**C22**	2012	m	15/11/2013	23/06/2014	221	2271	10.3
**C9**	2013	m	16/08/2013	23/01/2014	158	1469	9.3

Table S2: General information about GPS-tagged non-breeding ravens (ID) in France. Most individuals were captured on different days. The data collection for some ravens already stopped before end of November 2016 due to (i) technical reasons in one case (individuals were observed with the GPS logger but no data were transmitted), (ii) removal of the GPS-logger by the raven (n=3) and (iii) death through predation (n=1), illegal hunting (n=1) and unknown (n=1).

ID	fledged in	transloc-ated [km]	date of tagging	last GPS fix	# days with data	# GPS fixes	mean # of fixes per day
**f10.1**	2015	0	31/08/2015	27/11/2015	89	1126	12.7
**f10.2**	2015+	0	30/11/2015	30/11/2016	367	4168	11.4
**f24**	2015	0	31/08/2015	30/11/2016	452	4658	10.3
**f25**	2014	0	09/09/2015	08/10/2016	396	4731	11.9
**f29.2**	2015	0	26/07/2016	30/11/2016	128	1524	11.9
**f43**	2016	0	24/09/2016	30/11/2016	68	842	12.4
**f45**	2016	0	24/09/2016	30/11/2016	68	858	12.6
**fG12**	2014	0	10/04/2015	29/11/2016	577	4600	8.0
**fG16**	2015	0	30/11/2015	30/11/2016	367	4619	12.6
**fG17**	2015	0	26/07/2016	30/11/2016	128	1608	12.6
**f39**	2015	24.8	31/10/2015	07/05/2016	190	2332	12.3
**f23**	2015	40	24/09/2015	30/11/2016	434	5448	12.6
**f40.1**	2015	62.5	31/10/2015	19/12/2015	50	583	11.7
**f40.2**	2015	62.5	06/01/2016	30/11/2016	330	4329	13.1
**f26**	2015	160	14/08/2015	20/11/2016	465	5377	11.6
**f27**	2014	160	03/08/2015	04/12/2015	123	1589	12.9
**f29.1**	2015	160	10/12/2015	07/05/2016	150	1848	12.3
**f28**	2015	240	01/09/2015	30/11/2016	457	5504	12.0

Table S3: Association matrix for the percentage of time steps, when two individuals (C6 – C22) were within 100m in the Austrian study area.

	C6	C8	C9	C10	C11	C12	C21	C22
**C5**	3.1	0	0	0	20.9	13.8	0.7	0
**C6**	-	0	0	0	0	0	0	0
**C8**	-	-	0	0	0	0	0	0
**C9**	-	-	-	30.6	7.4	0	10.6	6.5
**C10**	-	-	-	-	11.0	0	6.6	21.1
**C11**	-	-	-	-	-	6.7	2.5	0.3
**C12**	-	-	-	-	-	-	0	0
**C21**	-	-	-	-	-	-	-	12.4

Table S4: Association matrix for the percentage of time steps, when two individuals were within 100m in the French study area. Asterisks mark combination, where individuals were not GPS-tagged at the same time. The colored lines group into different translocation distances: left of the blue = no translocation, between blue and red line = short distance, between red and purple line = medium distance, right of the purple line = large distance (see also Table S2).

	f10.2	f24	f25	f29.2	f43	f45	fG12	fG16	fG17	f39	f23	f40.1	f40.2	f26	f27	f29.1	f28
**f10.1**	*	6.8	4.6	*	*	*	0	*	*	13	0	0	*	0	0	*	0
**f10.2**	-	12.6	11.3	6.2	2.3	4.8	4.1	1.3	7.7	5	0	0	8.4	0	0	0	0
**f24**	-	-	13	12.8	0.4	4.6	4.5	2.4	14.9	6.2	0	0	13.3	0	0	0	0
**f25**	-	-	-	12.8	6	11	4.3	1.9	16.1	5.5	0	0	14.4	0	0	0	0
**f29.2**	-	-	-	-	1.3	10.8	2.3	0	19.1	*	0	*	4.6	0	0	*	0
**f43**	-	-	-	-	-	1.1	0	0	0.4	*	0	*	0	0	*	*	0
**f45**	-	-	-	-	-	-	0	0	11.8	*	0	*	0.6	0	*	*	0
**fG12**	-	-	-	-	-	-	-	0.1	4.3	0	0	0	7	0	0	0	0
**fG16**	-	-	-	-	-	-	-	-	0	3	0	0	0.3	0	0	0.8	0
**fG17**	-	-	-	-	-	-	-	-	-	*	0	*	7	0	*	0	0
**f39**	-	-	-	-	-	-	-	-	-	-	0	0	0.6	0	0	0	0
**f23**	-	-	-	-	-	-	-	-	-	-	-	0	0	0	0	0	0
**f40.1**	-	-	-	-	-	-	-	-	-	-	-	-	*	0	*	0	0
**f40.2**	-	-	-	-	-	-	-	-	-	-	-	-	-	0	*	0	0
**f26**	-	-	-	-	-	-	-	-	-	-	-	-	-	-	9.5	1.5	0
**f27**	-	-	-	-	-	-	-	-	-	-	-	-	-	-	-	*	0
**f29.1**	-	-	-	-	-	-	-	-	-	-	-	-	-	-	-	-	0

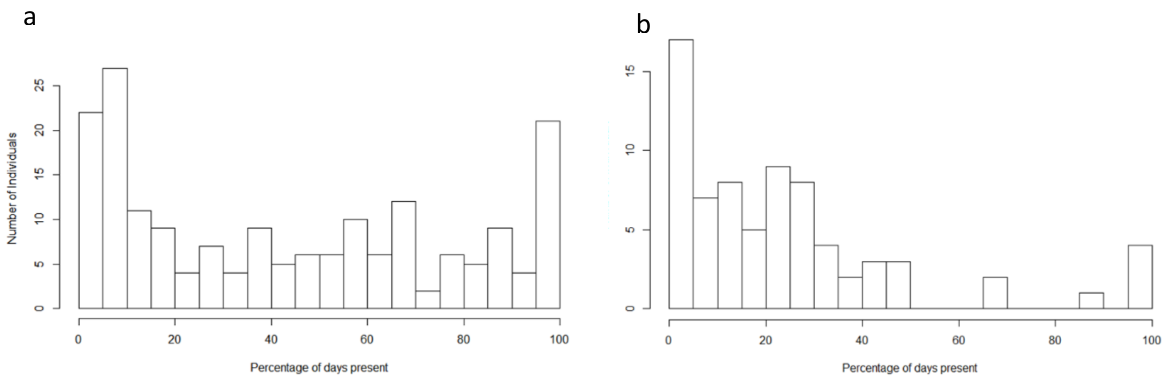

Figure S1: The histograms show for each marked raven the percentage of days observed between trapping and last observation at a) the Austrian study site (n=185 individuals) and b) the Italian study site (n=52 individuals).
